# Antibodies against Schmallenberg virus detected in cattle in the Otjozondjupa region, Namibia

**DOI:** 10.4102/jsava.v89i0.1666

**Published:** 2018-08-16

**Authors:** Umberto Molini, Andrea Capobianco Dondona, Renate Hilbert, Federica Monaco

**Affiliations:** 1 Department of Biotechnology, Central Veterinary Laboratory, Namibia; 2 Department of Diagnosis and Surveillance of Exotic Viral Diseases, Istituto Zooprofilattico Sperimentale. dell’Abruzzo e del Molise ‘G. Caporale’, Italy; 3 Directorate of Veterinary Services, Grootfontein, Namibia

## Abstract

Several ruminant species have been shown to be susceptible to Schmallenberg virus (SBV), but adult animals usually recover after showing mild or no clinical signs. However, transplacental infection can occur and lead to abortion, malformations and stillborn lambs, calves and goat kids. During November and December 2014, malformations were observed in 11 stillborn calves from two farms in the north-eastern region of Namibia. Blood samples were collected from 9 of the 11 cows that delivered stillborn and malformed calves. All these animals tested negative for Rift Valley fever, bovine viral diarrhoea and infectious bovine rhinotracheitis and were serologically positive for bluetongue virus, SBV and epizootic haemorrhagic disease virus. Clinical findings and serological results suggested that SBV may be circulating in Namibia.

## Introduction

Schmallenberg virus (SBV), a recently discovered *Orthobunyavirus* within the *Bunyaviridae* family, is a member of the Simbu serogroup and is closely related to Akabane and Shamonda viruses (Rasmussen et al. 2012). Several ruminant species have shown to be susceptible to SBV (European Food Safety Authority [EFSA] 2012), but adult animals usually recover after showing mild or no clinical signs. However, transplacental infection can occur and lead to abortion, stillborn and malformed lambs, calves and goat kids (Beer, Conraths & van der Poel 2013). Schmallenberg virus emerged in late 2011 in Germany and the Netherlands and it then spread to at least 20 different countries in Europe (EFSA 2012). The circulation of SBV in Africa was first reported in 2014 in domestic ruminants in Mozambique (Blomström et al. 2014), but limited information is available on its distribution in other countries in the continent.

## Materials and methods

During November and December 2014, morphologic anomalies were observed in 11 stillborn calves from two farms in the Grootfontein and Otavi districts in the north-eastern region of Namibia. These calves showed a variety of malformations, including arthrogryposis, abnormal curvature of the vertebral column, ankylosis of joints and severe muscle atrophy. Blood samples were collected from 9 of the 11 cows that delivered stillborn and malformed calves. Separated sera were stored at -20°C and sent to Istituto Zooprofilattico Sperimentale dell’Abruzzo e del Molise in Italy for serological investigations. Serological screening for SBV was performed using the ID Screen^®^ Schmallenberg virus Competition Multi-species ELISA (ID-vet, Grabels, France) according to manufacturer’s instructions, whereas the serum neutralisation test was performed using the BH80/11–4 isolate (provided by the Friedrich-Loeffler-Institut, Isle of Riems, Germany). Antibody detection for bluetongue virus (BTV) was performed using the competitive ELISA described by Lelli et al. (2013). A commercially available competitive ELISA kit (LSIVet^tm^ Ruminant EHDV – Serum, Life Technologies, Carlsbad, CA, USA) was chosen to test for antibodies against epizootic haemorrhagic disease virus (EHDV) according to the manufacturer’s instructions, while the serum neutralisation test for BTV and EHDV was performed using the method described by Gard and Kirkland (1993).

## Results

**FIGURE 1:**
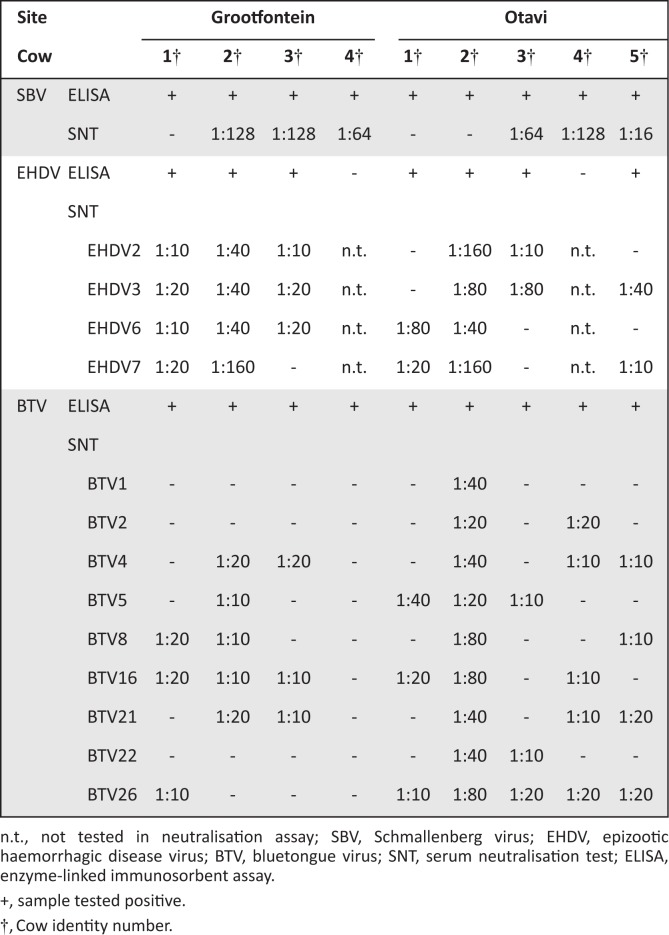
Details of serological results for antibodies to Schmallenberg virus, epizootic haemorrhagic disease virus and bluetongue virus in nine cows.

Serological tests confirmed antibodies to SBV, BTV and EHDV on both farms located in the Otjozondjupa region (Figure 1). All animals tested negative for Rift Valley fever, bovine viral diarrhoea and infectious bovine rhinotracheitis.

## Discussion

The brains of malformed calves were not collected. It was therefore not possible to perform either reverse transcriptase polymerase chain reaction or virus isolation on tissues from stillborn animals. Transplacental infection in cattle is possible only when the first placentome is present (within 30 days after conception), and because calves were conceived between February and June, this is the only window (between 30 and 150 days after conception) that may have led to porencephaly or hydranencephaly-micromyelia-arthrogryposis syndrome (Bayrou et al. 2014). The fact that animals got infected between February and June links infection to the rainy season (December to May), and this is consistent with the peak of the vector season when *Culicoides* are most active and when outbreaks of vector-borne diseases are often observed in the area (Capobianco Dondona et al. 2016). Clinical findings and serological results suggest that SBV may be circulating in Namibia. Cross-reactivity with other members of the Simbu serogroup cannot, however, be ruled out (Kinney & Calisher 1981). The ELISA chosen for this study detects antibodies against the nucleocapsid protein (N) of SBV, encoded by the sRNA segment, which has shown no cross-reactivity with Akabane or Rift Valley fever virus (Blomström et al. 2014). Although it is acknowledged that the sample size of nine animals is small and SBV was not isolated or the genome detected, cattle populations in Namibia are locally bred with very limited introduction of breeding animals from neighbouring countries. The presence of pure endemic breeds and cross-breeds, less susceptible to endemic diseases such as BTV and EHDV, strengthens the hypothesis that a different pathogen caused the outbreak. Neither embryo transfer nor artificial insemination was performed on the affected farms, and this supports the notion that SBV circulated in Namibia in 2014. Further investigations are needed to better understand the spread of SBV in Namibia and the role that midges and wildlife may play in the spread of the disease in the country. African isolates would furthermore help to determine the genetic relationship with European strains and to establish whether SBV originated in Africa and then spread to Europe or vice versa.
